# Quantitative Assessment of the Arm/Hand Movements in Parkinson’s Disease Using a Wireless Armband Device

**DOI:** 10.3389/fneur.2017.00388

**Published:** 2017-08-11

**Authors:** Sofija Spasojević, Tihomir V. Ilić, Ivan Stojković, Veljko Potkonjak, Aleksandar Rodić, José Santos-Victor

**Affiliations:** ^1^School of Electrical Engineering, University of Belgrade, Belgrade, Serbia; ^2^Mihailo Pupin Institute, University of Belgrade, Belgrade, Serbia; ^3^Institute for Systems and Robotics, Instituto Superior Técnico, Universidade de Lisboa, Lisbon, Portugal; ^4^Department of Neurology, Medical Faculty of Military Medical Academy, University of Defense, Belgrade, Serbia; ^5^Computer and Information Sciences Department, College of Science and Technology, Temple University, Philadelphia, PA, United States

**Keywords:** Parkinson’s disease, wireless sensors, arm/hand movements, bradykinesia, movement performance indicators

## Abstract

We present an approach for quantitative assessment of the arm/hand movements in patients with Parkinson’s disease (PD), from sensor data acquired with a wearable, wireless armband device (Myo sensor). We propose new *Movement Performance Indicators* that can be adopted by practitioners for the quantitative evaluation of motor performance and support their clinical evaluations. In addition, specific *Movement Performance Indicators* can indicate the presence of the bradykinesia symptom. The study includes seventeen PD patients and sixteen age-matched controls. A set of representative arm/hand movements is defined under the supervision of movement disorder specialist. In order to assist the evaluations, and for progress monitoring purposes, as well as for assessing the amount of bradykinesia in PD, a total set of 84 *Movement Performance Indicators* are computed from the sensor readings. Subsequently, we investigate whether wireless armband device, with the use of the proposed *Movement Performance Indicators* can be utilized: (1) for objective and precise quantitative evaluation of the arm/hand movements of Parkinson’s patients, (2) for assessment of the bradykinesia motor symptom, and (3) as an adequate low-cost alternative for the sensor glove. We conducted extensive analysis of proposed *Movement Performance Indicators* and results are indicating following clinically relevant characteristics: (i) adequate reliability as measured by ICC; (ii) high accuracy in discrimination between the patients and controls, and between the disease stages (support to disease diagnosis and progress monitoring, respectively); (iii) substantial difference in comparison between the left-hand and the right-hand movements across controls and patients, as well as between disease stage groups; (iv) statistically significant correlation with clinical scales (tapping test and UPDRS-III Motor Score); and (v) quantitative evaluation of bradykinesia symptom. Results suggest that the proposed approach has a potential to be adopted by physicians, to afford them with quantitative, objective and precise methods and data during clinical evaluations and support the assessment of bradykinesia.

## 1 Introduction

Contemporary approach to evaluation of the patient’s condition in Parkinson’s disease (PD), as well as assessment of the rehabilitation effectiveness, is based on the clinical assessment tools and evaluation scales, such as Hoehn and Yahr (HY) (1) and Unified Parkinson’s Disease Rating Scale (UPDRS) (2). However, although beneficial and commonly used, those scales are descriptive (qualitative), primarily intended to be carried out by a trained neurologist, and are prone to subjective rating and imprecise interpretation of patient’s performance.

Recent developments in the field of affordable sensing technologies have a potential to improve and support traditional evaluation techniques, aiming at defining quantitative movement indicators to assist practitioners and clinicians. Various types of wearable sensors have been proposed in the literature for the measurement and assessment of the arm/hand movements: accelerometers (3, 4), gyroscopes (5, 6), magnetic sensors (7, 8), force sensors (9, 10), and inertial sensors (11). However, these sensor systems only modestly contribute to the arm/hand movement assessment. Specifically, the use of one or two isolated sensors in motion acquisition restricts the movement quantification, due to the limited amount of the collected data.

More informative sensors are the ones that measure muscle activity, and the standard approach for obtaining the muscle activity information is the placement of the surface Electromyography (EMG) electrodes on the skin, which detect the electrical potential generated by muscles. The main drawback of the standard EMG electrodes is the wired connection with a device for EMG signal representation. Consequently, muscle activity tests are available only in the hospital environment. The analysis of the muscle activity is reported in some recent studies concerning PD (12–14). The authors in Ref. (12, 13) particularly observe the muscles’ behavior during deep brain stimulation. They report that Parkinson’s disease symptoms change the EMG signal properties and suggest that EMG analysis is able to detect differences between the deep brain stimulation settings. The authors in Ref. (14) use the EMG data, along with the readings from the accelerometer, to successfully differentiate essential tremor from Parkinson’s disease. However, all these studies collect the EMG data using surface electrodes relying on the wired system.

The authors have suggested many different features to characterize the EMG signals in the time domain (13–21) and frequency domain (15, 16, 19, 21). The two most common approaches for the EMG signal analysis are the wavelet transform (14, 21) and the window approach (15, 19). In our study, we have adopted the window approach and the features suggested in the literature that emphasize the amplitude characteristics of the EMG signal. Such choice has been convenient for our case as it will be explained in detail in the Results section.

In our previous studies (22, 23), we have used a vision-based sensor (Kinect device) to quantify full-body movements (gait and large-range upper body movements) and a sensor glove (CyberGlove II device) to quantify hand movements of Parkinson’s patients. We proposed novel scores called Movement Performance Indicators that were extracted directly from the sensor data and quantify the symmetry, velocity, and acceleration of the movement of different body/hand parts. Our approach for the hand movement characterization, based on the sensor glove data, has demonstrated significant results and ability to support the diagnosis and monitoring evaluations in PD (23). Still, due to the high cost, it does not fit into our concept of a low-cost rehabilitation system for movement analysis. Another limitation arises from the right-hand design of the sensor glove device. This implies that only right-hand movements can be tested; and hence, only right side affected patients are taken into account. Consequently, left–right side analysis cannot be conducted as an important indicator of the disease progression.

In this study, we focus on quantification of the arm/hand movements from measurements acquired with a wireless wearable armband device—the Myo sensor,^1^ in order to investigate whether the armband sensor can assess fine movements and be used as a suitable alternative to the sensor glove. This device is placed on the forearm and outputs Electromyography (EMG) data from eight channels. EMG data provide insight into the muscle activity information. Impaired muscle activity and restriction of motor functions are common characteristics of PD. The armband device contains also three-axis accelerometer and three-axis gyroscope, which output acceleration and angular velocity information (Inertial Measurement Unit (IMU) data), respectively.

The accelerometer and gyroscope have been widely tested in studies related to PD and showed significant potential toward quantification of PD symptoms (14, 24–26). The authors in Ref. (24) use accelerometers, while the authors in Ref. (26) use both, accelerometers and gyroscopes, to observe the gait characteristics in PD patients. They state that freezing of the gait episodes can be detected using sensor data, along with the feedback about gait performance. The study (25) focuses on the quantification of bradykinesia from finger-tapping movement using two gyroscopes placed on the fingers. Although the results of bradykinesia quantification using gyroscope data are promising, the analysis is limited to one movement and two sensors. The overall conclusion is that signals from accelerometer and gyroscope demonstrate meaningful patterns in the patient’s movements and reveal the presence/intensity of the disease motor symptoms. Like in the case of EMG signals, we concentrate on the signal features from accelerometer and gyroscope that take into account the signal amplitude characteristics.

The wireless armband device has been launched very recently and only a few conceptual studies report some preliminary results concerning its inclusion into medical protocols (27–29). However, to the best of our knowledge, it has not been previously used in any study regarding the quantification of the arm/hand movements in PD assessment.

Our study overcomes the scope of conceptual studies published so far, by introducing the comprehensive processing modules and interpretation of the sensor measurements from armband device. We propose new scores for the arm/hand movement characterization denoted as Movement Performance Indicators (hereinafter, MPIs). The MPIs are intended to support diagnosis and monitoring evaluations, as well as the assessment of the motor symptoms, with a special emphasis on bradykinesia. The MPIs we propose are built upon both domain-specific knowledge (provided by movement disorder specialist), as well as data analysis. They are primarily designed in accordance with clinically relevant aspects and tested toward official clinical tests and scales. We thus propose an affordable, reliable, and portable sensor system along with an approach for movement quantification, with the potential to be used as a support for the conventional motor performance evaluations and the possibility of home rehabilitation.

In this article, we present extensive experiments and analysis conducted to address the following aspects: (1) quantitative evaluation of the arm/hand movements of Parkinson’s patients, (2) objective assessment of bradykinesia motor symptom, and (3) investigation whether the armband sensor can be an adequate low-cost alternative for the sensor glove, due to its high cost. Aspects addressed in (1) and (2) are worth to be investigated in the treatment of Parkinson’s disease, but their direct assessment is not possible considering the limited resources and standard techniques used by doctors.

## 2 Materials and Methods

### 2.1 Participants

Seventeen Parkinson’s disease patients (age = 63.5 ± 8.3,^2^ disease duration = 4.7 ± 2.5, HY^3^ disease stage = 2.59 ± 0.93, UPDRS-III^4^ = 31.82 ± 15.43 during ON-period) have been tested in this study. Patients are examined during their first ON-period in the morning. For ten patients, the right hand is affected by the disease, while seven patients have the left hand affected. A control group is formed by sixteen age-matched volunteers without any history of neurological or movement disorder. All subjects have been examined under the same conditions and instructed by a neurologist and therapists. This study was approved by the local ethics committee according to the Declaration of Helsinki. After the experimental procedures were explained, all subjects signed written informed consent forms.

### 2.2 Experimental Protocol

**Figure 1:**
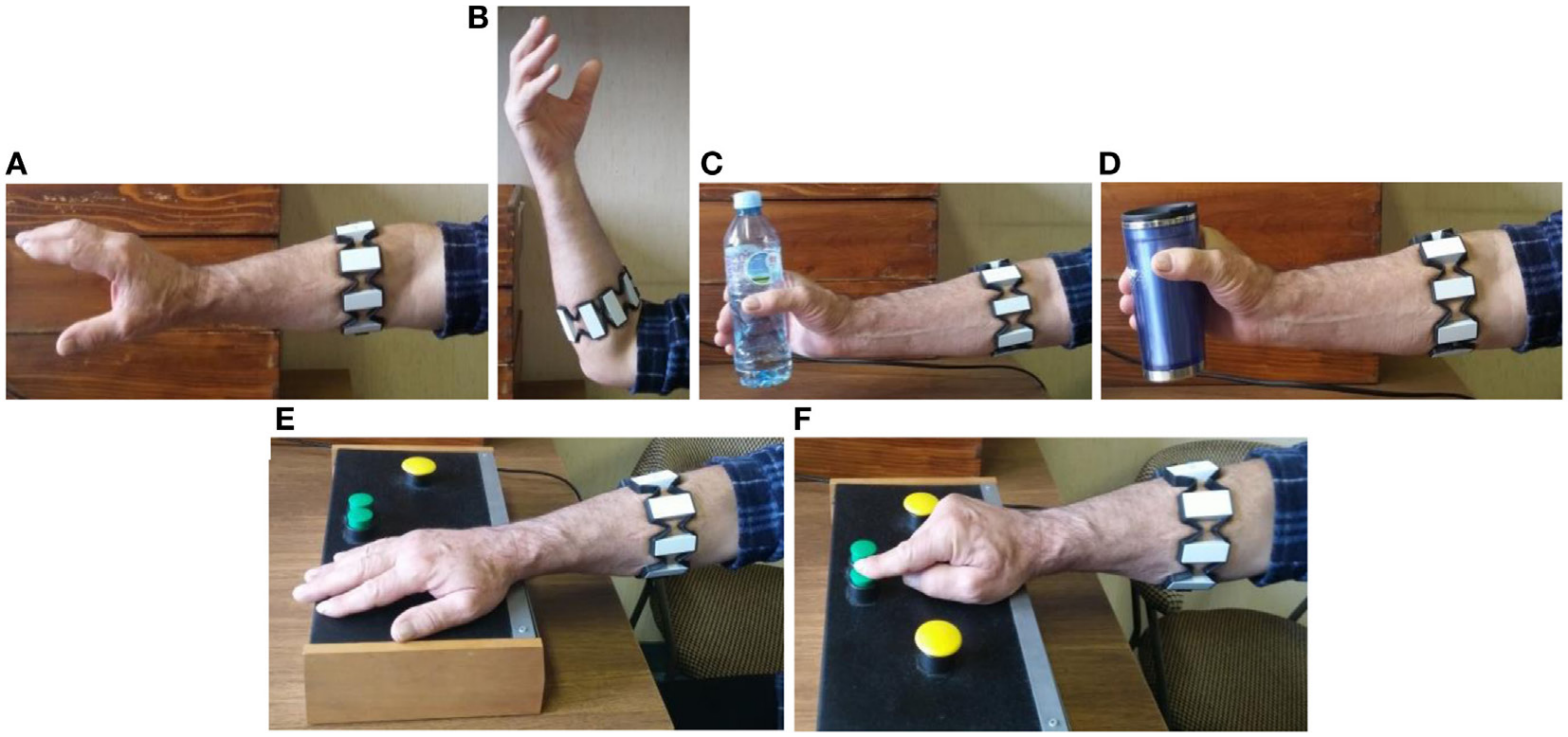
Movements acquired according to the experimental protocol: RH-EE **(A)**, RH-EF **(B)**, GPP-EL **(C)**, GPP-HL **(D)**, TT-P **(E)**, and TT-D **(F)**.

The experimental protocol, designed by the movement disorder specialists (Table 1; Figure 1), includes six exercises performed with the left and right hand: four arm/hand movements and two tapping test movements, well-established experimental paradigm designed for bradykinesia assessment (30). The tested movements are chosen to closely reflect the patient’s activities of daily living that engage forearm muscles. The movements have been performed with the left and right hand, respectively, and acquired using the armband sensor. The subjects were instructed to perform the movements as fast as possible.

**Table 1:** Acquired movements according to the experimental protocol and their acronyms used in the article.

	Movements acquired according to the experimental protocol	Acronyms used in the article
1.	**R**otation of the **H**and with **E**lbow **E**xtended	RH-EE
2.	**R**otation of the **H**and with **E**lbow **F**lexed at 90°	RH-EF
3.	Object **G**rasping, **P**ick and **P**lace in the case of **E**asy **L**oad	GPP-EL
4.	Object **G**rasping, **P**ick and **P**lace in the case of **H**eavy **L**oad	GPP-HL
5.	The **P**roximal **T**apping **T**ask	TT-P
6.	The **D**istal **T**apping **T**ask	TT-D

The medical procedure adopted in PD analysis includes a set of movements/exercises, in order to allow doctors to make a qualitative evaluation of the disease stage and progress. The first two exercises emulate the bulb screwing/unscrewing in two variations: *Rotation of the Hand with Elbow Extended* (**RH-EE**, Figure 1A) and *with Elbow Flexed* at 90° (**RH-EF**, Figure 1B). Those movements were acquired during the period of 10 s. The following two exercises relate to the object *Grasping, Pick and Place in the case of Easy Load* (**GPP-EL**, Figure 1C) and *Heavy Load* (**GPP-HL**, Figure 1D). Those movements were repeated five times. The last two exercises represent the tapping test. The test consists of the proximal and distal tapping tasks using a specially designed board as the one proposed in Ref. (30). *The Proximal Tapping Task* refers to the alternate pressing of two large buttons located 20 cm apart with the palm of the hand, during the 30 s interval (**TT-P**, Figure 1E). *The Distal Tapping Task* is related to the alternate pressing of two closely located buttons (3 cm apart) with the index finger while the wrist is fixed on the table during 30 s (**TT-D**, Figure 1F). The acquired data consist of: (i) EMG data from 8 channels (sensor data rate 200 Hz) and (ii) three-axes IMU data—acceleration and angular velocity (sensor data rate 50 Hz).

**Figure 2:**
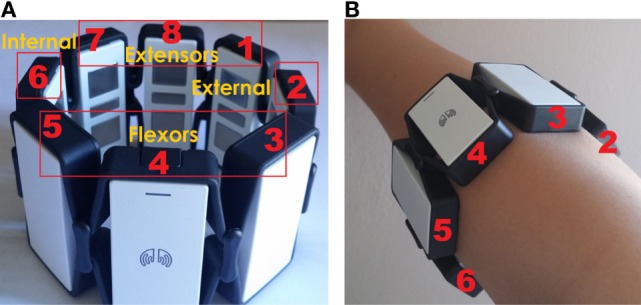
Labeled channels of the armband sensor **(A)** and armband sensor placement on the right hand during experiments **(B)**.

The armband sensor consists of eight EMG channels labeled as shown in Figure 2A. During the experiments, the sensor was placed in the same position for every subject (Figure 2B, right hand). It can be seen that for the right-hand channels 3, 4, and 5 cover the upper forearm (extensors muscles), channels 7, 8, and 1 are placed on the lower forearm (flexors muscles), channel 2 covers the external forearm muscles, while the channel 6 is placed on the internal forearm muscles. As for the left hand, extensors and flexors are covered with the same groups of channels, while the channels 2 and 6 are replaced between internal (channel 2) and external (channel 6) forearm muscles.

### 2.3 Data Processing

In this section, we explain the design of the seven basic measurements, based on which MPIs are grounded. The choice of the basic measurements is based on the properties of the sensor signals in the time domain (signal amplitude). The readings from the EMG electrodes, as well as outputs from an accelerometer and gyroscope, are used for movement characterization.

Before the basic measurements calculation, the signals are preprocessed to remove the measurement noise and for performing temporal segmentation. In our experiments, all signals were filtered with regular Butterworth low pass filter. Cutoff frequencies and order of the filter were chosen in accordance with the signal sampling rate and the frequency characteristic of the meaningful signal content. EMG signals are filtered using 4th order filter with cutoff frequency of 20 Hz. As for the accelerometer and gyroscopes signals, the cutoff frequency is set to 5 Hz and filter order to 3. The segmentation procedure is required in order to remove the non-informative signal parts at the beginning and at the end of the signals. For this purpose, the threshold based on the signal energy in the time domain has been adopted (0.4 times the maximum signal energy).

**Figure 3:**
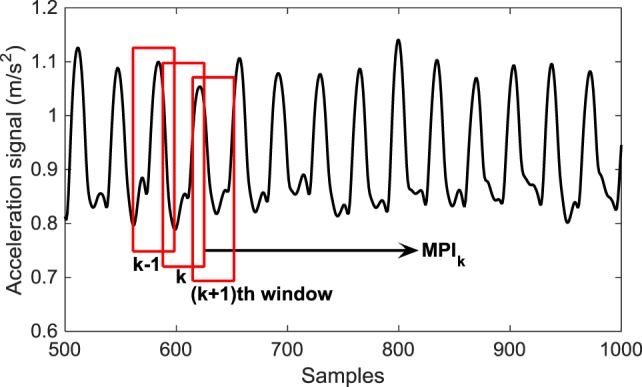
Window approach for basic measurements extraction illustrated for the case of the acceleration signal.

Since the EMG signals are highly non-stationary, the most common approach for the processing of the EMG signals is the window approach (15, 19). This method implies the temporal segmentation of the signal into sliding windows and calculating the particular value of basic measurements for each separate window (Figure 3). The same technique has been applied to the signals obtained from the accelerometer and gyroscope. The main benefit of the window analysis is to characterize the temporal evolution of basic measurements during the movement.

Different lengths of the window and overlapping segment are tested and the results were not sensitive to those choices of the length. We set the window length to 200 ms for EMG signals and 800 ms for signals from accelerometer and gyroscope. The length of the overlapping segment usually amounts 25–50% of the window length as suggested in Ref. (15, 19). We choose the length of the overlapping segment as 25% of the window size, hence 50 ms for EMG signals and 200 ms for signals from accelerometer and gyroscope.

#### 2.3.1 Quantification of the EMG Signals

Various measurements have been proposed in the literature for characterization of the EMG signal (15–19). Our choice of suitable basic measurements from EMG signal relies on the signal amplitude properties; hence, we tested amplitude-based measurements that are most often used in the literature. Thus, we have quantified obtained EMG signals using the Mean Absolute Value (**Emg-mav**) (1), Variance (**Emg-var**) (2), and Waveform Change (**Emg-wc**) (3). In equations (1)–(3), *W_n_* represents the window length, expressed in signal samples.

(1) EmgMAV=1Wn∑t=1Wn|EMG(t)|

(2) EmgVAR=1Wn∑t=1Wn EMG(t)2

(3) EmgWC=∑t=1Wn−1|EMG(t+1)−EMG(t)|

#### 2.3.2 Quantification of the Signals from an Accelerometer and Gyroscope

The accelerometer (ACC) and gyroscope (GYRO) signals are quantified using the same time-window approach as for EMG signals. The choice of basic measurements is different, in accordance with the signal characteristics and the properties of its transformations (such as signal derivative). The accelerometer and gyroscope signals are not processed in their original form. Instead, the basic measurements are extracted from their time-derivatives since the signal derivative enlarges the differences between controls and patients. Extracted basic measurements are *Simple Square Integral* (**SSI**) and *Range* (**RAN**), given by equations (4) and (5), respectively, where $\dot{x}(t)$ represents the accelerometer or gyroscope signal derivative.

(4) (Acc/Gyro)SSI=∑t=1Wn x˙(t)2

(5) (Acc/Gyro)RAN=max(x˙(t))−min(x˙(t)), t∈{1,Wn}

The above specified basic measurements are directly related to the signal amplitude—larger amplitude indicates larger value of basic measurements defined by equations (4) and (5).

### 2.4 Data Analysis

The MPIs are designed to emphasize the largest differences between patients and controls. We investigate whether the EMG data from particular channels are more discriminative than others. The comparative statistical analysis between patients and controls across six collected movements and eight EMG channels has been conducted using Wilcoxon rank sum test. In addition, we consider the difference of the group mean values as an indicator of the difference between groups of interest.

The same statistical test is conducted for accelerometer and gyroscope sensor data. They have three axes and depending on the particular movement, the data from one axis are more relevant than the data from the remaining two. Consequently, for each movement, corresponding axis of interest is adopted based on the statistical analysis using Wilcoxon rank sum test and comparison between group mean values.

#### 2.4.1 Reliability Analysis

In order to test the reliability of the extracted MPIs, the split-half method for reliability analysis (31) has been applied. The split-half method divides the conducted tests into two parts and correlates the scores on one-half of the test with scores on the other half of the test. Thus, the split-half method estimates the reliability based on the repetitions inside the same trial. Reliability of the extracted MPIs is assessed using *Intraclass Correlation Coefficient* (**ICC**) (31). ICC has a value inside range [0–1], whereby the values closer to 1 indicate higher reliability.

#### 2.4.2 Dimensionality Reduction

Finding lower dimensional representations which still preserve the most relevant information contained in the original data is key for many machine learning and data mining applications. It results in reduced data needs, reduced computational cost for algorithms, and often even increases the predictive performance of the learned models. Therefore, we have used two popular approaches for dimensionality reduction and feature selection, LDA (32) and LASSO regression (33), to find most relevant MPIs. LDA is a dimensionality reduction approach which finds the most discriminative principal components (linear combination of features), but can also rank the features by their importance. LASSO regression performs feature selection by assigning zero weights to less relevant features, giving them zero influence on the targeted outcome. Theoretically, the LASSO regression is more adequate to non-Gaussian type of data than LDA, but in practice they have similar predictive performance. Both algorithms have the same computational complexity, cubic in the number of features (*O*(*k*^3^)) and linear in the number of examples (*O*(*k*^3^*n)), where *k* is the number of features and *n* is the number of examples.

#### 2.4.3 Classification

We want to investigate how designed MPIs can be used to differentiate between the groups of interest. We analyze two distinct classification problems in order to support the diagnosis (patients against controls) and progress monitoring (disease stages). The diagnosis task is posed as discriminating the PD patients from the healthy controls, based on the measured values of MPIs, which is a well-known binary classification problem. We define the monitoring task as discerning among the three severity stages in PD patients, which is the multiclass classification problem. Multi-class disease stage classification problem we reduced to three simple binary classification problems, one for each stage, in a common “one vs all” manner (34).

To obtain the desired classifiers for diagnostic and monitoring purposes, we employed six common classification approaches: Logistic Regression, Decision Trees, Support Vector Machines (with RBF kernel), K-nearest neighbors (with number of nearest neighbors *k* = 10), Naive Bayes, and Neural Networks (multilayer perceptron with two hidden layers containing four nodes each).

#### 2.4.4 Comparison between Right and Left Side

To investigate which MPIs illustrate the differences in the performance of the left and right hand at patients and similar performance of the both hands in controls, statistical comparison has been performed. The choice of statistical tests depends on the data distribution. We performed the Kolmogorov-Smirnov test to assess the normal distribution hypothesis. The test rejected the normal distribution hypothesis with a 0.05 significance level. Consequently, two-sided Wilcoxon rank sum test is applied between the MPI values obtained with the left and right hand. There are forty-two MPIs in total for each hand—seven different MPIs for six movements. Three groups of interest have been considered (patients with the right side affected, patients with the left side affected and controls). For the disease stage analysis, both groups of the left and right side affected patients are additionally divided into the first three stage groups according to the Hoehn and Yahr scale (HY) (1).

The corresponding MPI is considered as relevant for the left–right side analysis between patients and controls if it satisfies the following conditions: (i) patients group: (a) if the difference between the MPI values for the left and right hand is statistically significant (*p* < 0.05) and (b) the left-hand MPI values are larger than the right-hand MPI values (for the right side affected patients) and the opposite for left-side affected patients and (ii) controls: if the difference between the MPI values for the left and right hand is not statistically significant (*p* > 0.05).

The same statistical tests were conducted for the left–right side analysis between disease stages. Statistical investigation is based on the following conditions: (i) the difference between the MPI values of the left and right hand is statistically significant (*p* < 0.05); (ii) the left-hand MPI values are larger than the right-hand MPI values (for the right side affected patients) and the opposite for left-side affected patients; and (iii) MPI values decrease with more severe disease stage, while their differences between the left and the right hand increase.

#### 2.4.5 Correlation Analysis

The correlation analysis is carried out between the proposed MPIs and tapping test (30) and UPDRS-III clinical scale (2). The tapping-test outcomes and UPDRS-III values are obtained as a result of a neurologist’s evaluation. The tapping test consists of two tapping tasks—proximal and distal tapping task explained in the Section 2.2. In the case of UPDRS-III, we take into account the general UPDRS-III score (items 18–31 of UPDRS scale (2)) and UPDRS-III subscore related to the examination of the bradykinesia in the hand movements (items 23–25 of the UPDRS scale (2)).

Correlations were calculated using Spearman correlation coefficient *ρ* (higher values of *ρ* indicate better correlation), along with the *p*-value. If the correlation coefficient *ρ* is in the range [0.5–1] and *p*-value less than 0.05, the corresponding MPI is correlated with the tapping test (positive correlation). On the other side, the correlation coefficient *ρ* between −1 and −0.5 and *p*-value less than 0.05, indicate the correlation of the particular MPI with UPDRS-III scale (negative correlation).

## 3 Results

### 3.1 Preliminary Comparison between PD and Controls

**Figure 4:**
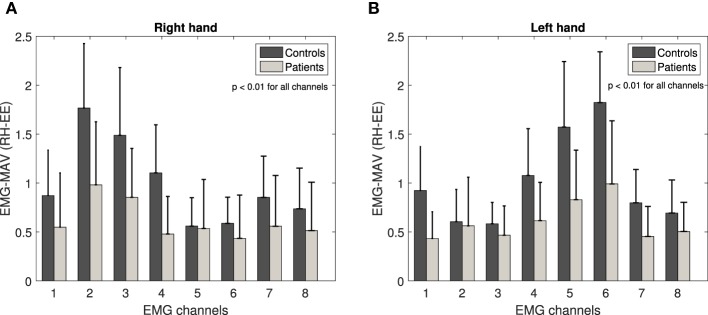
Emg-mav basic measurement across eight EMG channels for RH-EE movement: right hand **(A)** and left hand **(B)**. Channels 2 (right hand) and 6 (left hand) underline the largest mean value difference between controls and patients.

Figure 4 illustrates the mean absolute value and the standard deviation graph of Emg-mav basic measurement (1) calculated for patients and controls across eight EMG channels for RH-EE movement. The results underline the largest mean value differences between controls and patients on the channel 2 in the case of the right-hand movements and channel 6 for the left-hand movements.

Figure 2 shows that those electrodes cover the same group of external forearm muscles in the case of both hands. In addition, channels 3 and 4 (right-hand movements) and channels 4 and 5 (left-hand movements) highlight the large differences, as well (external and upper flexor muscles). The data from all channels demonstrated statistically significant difference between patients and controls (*p* < 0.01). However, in the following analysis, we take into account channels that emphasize the largest difference between group mean values and consequently, the extraction of the basic measurements has been performed only for the signals from channel 2 for the right-hand movements and from channel 6 for the left-hand movements. The same results are confirmed for remaining EMG basic measurements (2 and 3) and all other collected movements.

**Figure 5:**
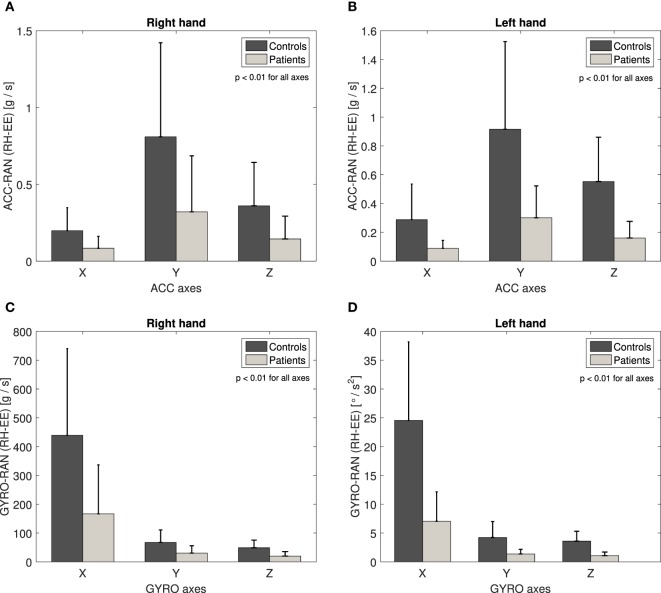
Acc-ran basic measurement across three axes for RH-EE movement: right hand **(A)** and left hand **(B)**. *Y*-axis underline the largest mean value difference between controls and patients. Gyro-ran basic measurement across three axes for RH-EE movement: right hand **(C)** and left hand **(D)**. *X*-axis underline the largest mean value difference between controls and patients.

Figure 5 illustrates the mean absolute value and the standard deviation graph of Acc-ran and Gyro-ran basic measurement (5) calculated for patients and controls across three axes for RH-EE movement. The results underline the largest mean value differences between controls and patients on the *Y*-axis for Acc-ran and on the *X*-axis for Gyro-ran in the case of both, right- and left-hand movements. The same analysis is performed for the other ACC and GYRO basic measurement (4) and all other collected movements. In contrast to EMG channels, the axis of interest for ACC and GYRO basic measurements is different across movements, but for the particular movement, the axis of interest is the same for right and left-hand movements. The data from all axes demonstrated the statistically significant difference between patients and controls (*p* < 0.01). However, in the following analysis, for each movement, we take into account the axis that emphasizes the largest difference between group mean values.

In total, we have extracted seven basic measurements (Table 2) for each movement. We characterize twelve movements—six different movements (Table 1 and Figure 1) were performed by both left and right hand. Consequently, based on the seven basic measurements calculated for each movement, we obtained a total set of 84 Movement Performance Indicators (MPIs) for all movements (seven basic measurements times twelve movements). The design of these MPIs was grounded on the information provided by neurologists and therapists with the goal of delivering quantitative information about subject’s performance. In the following sections, we will reveal which MPIs are the most relevant and informative, from the viewpoint of the particular clinical aspects.

**Table 2:** Calculated basic measurements.

	Calculated basic measurements	Acronyms used in the article
1.	**M**ean **A**bsolute **V**alue from **EMG** signal	Emg-mav
2.	**Var**iance from **EMG** signal	Emg-var
3.	**W**aveform **C**hange from **EMG** signal	Emg-wc
4.	**S**imple **S**quare **I**ntegral from **Acc**elerometer signal derivative	Acc-ssi
5.	**Ran**ge from **Acc**elerometer signal derivative	Acc-ran
6.	**S**imple **S**quare **I**ntegral from **Gyro**scope signal derivative	Gyro-ssi
7.	**Ran**ge from **Gyro**scope signal derivative	Gyro-ran

### 3.2 Reliability

**Figure 6:**
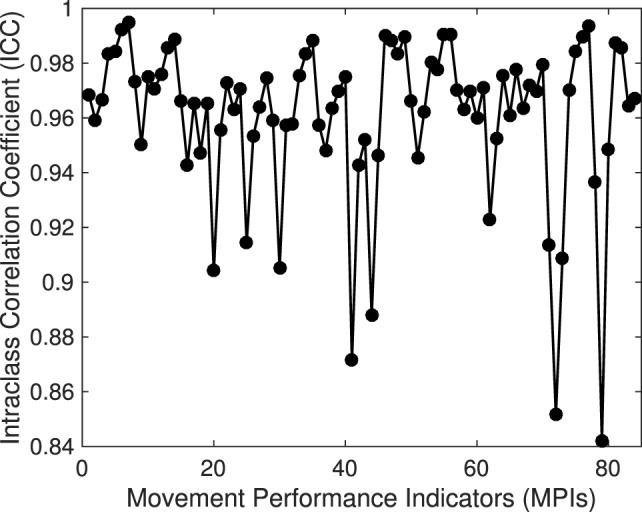
Intraclass Correlation Coefficient (ICC) across Movement Performance Indicators (MPIs).

The results of the reliability analysis indicate high reliability for all 84 MPIs, with ICC values in range [0.84–0.99], Figure 6.

### 3.3 Quantitative Assessment of Bradykinesia Symptom

**Figure 7:**
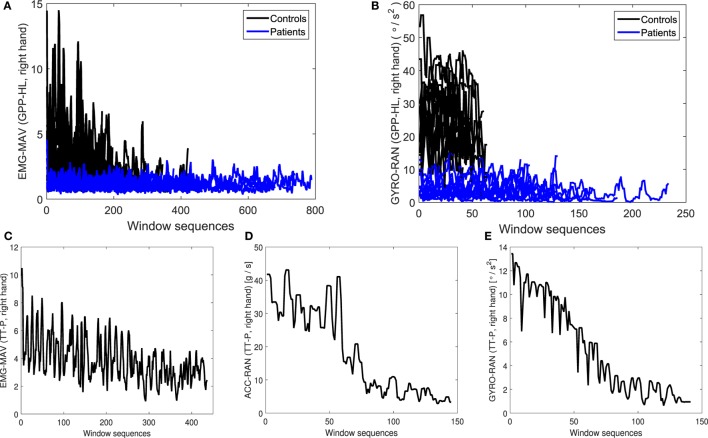
Illustration of bradykinesia symptom: temporal evolution of **(A)** EMG-mav and **(B)** GYRO-ran during GPP-HL movement. Patients performed slower movements than controls. Temporal evolution of **(C)** EMG-mav, **(D)** ACC-ran, and **(E)** GYRO-ran during TT-P movement for patient data. The values of basic measurements sequentially drop over time (bradykinesia “sequence effect”). * *Y*-axes are labeled in the form: basic measurement(s) (performed movement, hand).

In this section, we investigate whether our proposed MPIs can reveal the presence of bradykinesia symptom in patients. Two main properties of bradykinesia symptom are (1) slowness of the movements and (2) the progressive decrease in amplitude of sequential movements (so-called “sequence effect”). Figure 7 illustrates the bradykinesia pattern, relying on the designed MPIs. The difference in movement speed between patients and controls is demonstrated for GPP-HL movement since this movement was repeated five consecutive times during the experiment. Figure 7A shows the temporal evolution of the EMG-mav over window segments, for patients and controls, during the GPP-HL movement. The patients have demonstrated slower movements—they needed more time to perform five consecutive movements than controls.

In order to investigate the presence of “sequence effect” in the context of our proposed basic measurements, we analyze their evolution during the movement performance. We focus on the TT-P and TT-D movements since those movements are recorded in the period of 30 s, which enables enough sensor data for sequence effect analysis. Figure 7C demonstrates the temporal evolution of Emg-mav basic measurement during TT-P movement for right-hand affected patient (third disease stage according to Hoehn and Yahr (HY) (1)). The decrease of Emg-mav basic measurement over time is slow, but constant (Figure 7C). Such outcome suggests the presence of bradykinesia symptom.

The bradykinesia symptom is visible from the time evolution of ACC and GYRO basic measurements, as well. Figure 7B illustrates the temporal evolution of the Gyro-ran over window segments, for patients and controls, during the GPP-HL movement. The result is the same as in the case of EMG data—slower movements at patients are confirmed based on the evolution of Gyro-ran basic measurement over time. Bradykinesia “sequence effect” is confirmed based on the ACC and GYRO basic measurements, as well. However, the decreasing pattern is different from EMG data. ACC-ran values are significantly larger in the first-half period compared to the second-half period (Figure 7D). Finally, GYRO-ran basic measurement (Figure 7E) shows the constant and significant drop in values over time.

### 3.4 Dimensionality Reduction and MPIs Selection

**Figure 8:**
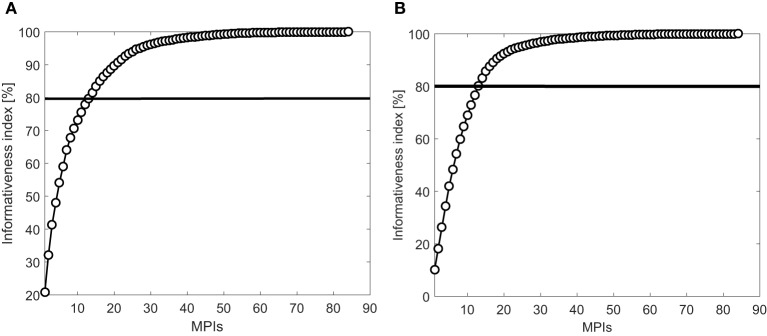
LDA Informativeness index: **(A)** patients–controls and **(B)** disease stages.

We applied Linear Discriminant Analysis (LDA) (32) to determine the most relevant MPIs for the decision-making process based on the clinical group parameter, between patients and controls (diagnosis support) and between disease stages (monitoring support). The implementation of the LDA method is based on the procedure described in detail in our previous research (23). Information index plots (Figures 8A,B) show the importance of the MPIs for classification tasks from the ones most important toward less important MPIs. The LDA method results that, for keeping 80% of information from the original data set, it is sufficient to select first 13 out of 84 MPIs for both conditions: patients/controls (Figure 8A) and disease stages (Figure 8B). The selected MPIs are listed in Table 3. Information index plots also demonstrate that some MPIs have the negligible impact on the classification tasks. After the first 50 MPIs, adding more MPIs will not bring significant information.

**Table 3:** The most relevant MPIs obtained by LDA approach and LASSO regression^a^ (bolded MPIs are the ones selected by both approaches).

	Patients/controls		Disease stage (HY)	
#	LDA	LASSO	LDA	LASSO
1.	Gyro-ssi TT-D-R	**Gyro-ssi TT-D-L**	**Emg-mav GPP-HL-R**	**Gyro-ssi TT-D-L**
2.	**Gyro-ssi TT-D-L**	**Emg-mav GPP-EL-L**	Emg-mav TT-P-R	**Emg-mav RH-EF-R**
3.	**Emg-mav GPP-EL-L**	**Emg-mav TT-D-R**	**Gyro-ssi TT-D-L**	**Gyro-ran TT-D-L**
4.	**Emg-mav GPP-HL-R**	**Emg-mav GPP-HL-R**	**Emg-mav RH-EF-R**	**Emg-mav GPP-HL-R**
5.	**Emg-mav TT-P-R**	**Gyro-ssi GPP-EL-L**	**Gyro-ran TT-D-L**	**Emg-mav GPP-EL-R**
6.	**Gyro-ssi GPP-EL-L**	Gyro-ran GPP-HL-L	**Emg-mav GPP-EL-R**	**Emg-mav TT-D-R**
7.	**Gyro-ran TT-D-L**	**Gyro-ssi GPP-HL-R**	**Emg-mav TT-D-R**	Emg-mav RH-EE-R
8.	Gyro-ssi GPP-HL-L	**Gyro-ran GPP-EL-L**	**Emg-mav RH-EE-L**	Gyro-ran GPP-HL-R
9.	**Gyro-ran GPP-EL-L**	**Gyro-ran TT-D-L**	Emg-mav GPP-HL-L	Gyro-ran TT-D-R
10.	Gyro-ran TT-D-R	**Emg-mav TT-P-R**	Gyro-ssi GPP-HL-L	Emg-mav TT-P-L
11.	Emg-mav GPP-HL-L	Emg-mav RH-EF-L	Emg-mav RH-EF-L	**Emg-mav RH-EE-L**
12.	**Emg-mav TT-D-R**	Gyro-ssi RH-EF-D	Gyro-ran GPP-HL-L	Gyro-ran TT-P-L
13.	**Gyro-ssi GPP-HL-R**	Gyro-ssi TT-P-D	Gyro-ssi TT-D-R	Emg-mav TT-D-L

*^a^MPIs are listed in the format MPI movement-hand (R-right or L-left)*.

In order to verify the results obtained by LDA, we have used the LASSO regression analysis (33), which performs both feature selection and regularization, in order to enhance the classification accuracy. Using the LASSO regression, the response variable (corresponding class of the interest—patients/controls or disease stage) is modeled as a linear combination of the MPIs (model parameters). The model parameters with strongest dependence of the response variable will have higher coefficients, while the coefficients corresponding to the less important parameters will weight toward zero. In such way, we select the most important model parameters (corresponding MPIs) according to the classification task of interest. Results of both techniques, LDA and LASSO, giving the 13 most relevant MPIs (out of 84 MPIs in total), and for the classification criterion between groups of interest, are listed in Table 3.

Table 3 shows that the 13 most relevant MPIs (out of 84 MPIs) are Gyro-ssi, Gyro-ran and Emg-mav extracted mostly from the movements of object grasping, pick and place (GPP-EL and GPP-HL) and tapping test movements (TT-P and TT-D). The list of the most relevant MPIs is not the same in case of LDA and LASSO regression, but the majority of representative MPIs are selected by both methods (marked as bold text in Table 3). Such result can be a consequence of the adjustment of regularization parameter *λ* ∈ [0.01–0.5] during Lasso regression. This parameter determines the strength of the penalty. As *λ* increases, more coefficients of the model are reduced to zero, hence more parameters (MPIs) are excluded from the model.

### 3.5 Classification: Diagnosis and Monitoring Evaluations

Classifiers were built for four tasks: (i) PD patients vs controls (PD vs C); (ii) stage I vs stages II and III PD; (iii) stage II vs stages I and III PD; and (iv) stage III vs stages I and II PD, and by using two sets of MPIs: (a) original (full) set of 84 MPIs and (b) set of 13 MPIs selected by LDA in Table 3. As a criterion of the classification success, the area under the ROC curve (AUC) is calculated (35). ROC curve represents the graph of the true positive rate (TPR) against the false positive rate (FPR). AUC is the calculated surface area under the ROC curve. AUC values that indicate high-performance classifiers are in the range [0.80–1]. The performance of each classifier is assessed in a (10-fold) cross-validation procedure, and the results are provided in Table 4 in form of a *mean (standard deviation)* calculated from 10-folds.

**Table 4:** Performance of six classification approaches in diagnostic and monitoring tasks for two sets of MPIs.

	Original (Full) set (84 MPIs)				Selected subset (13 MPIs—LDA)			
Classifier	PD vs C	Disease stages			PD vs C	Disease stages		
		I vs. II and III	II vs. I and III	III vs. I and II		I vs. II and III	II vs. I and III	III vs. I and II
Logistic regression	1 (0)	1 (0)	1 (0)	1 (0)	0.9967 (0.0034)	0.9942 (0.0088)	0.8969 (0.0569)	0.9961 (0.0074)
Decision trees	0.9905 (0.0114)	0.9670 (0.0286)	0.9499 (0.0582)	0.9649 (0.0441)	0.9823 (0.0091)	0.9542 (0.0504)	0.8840 (0.1074)	0.9308 (0.0344)
Support vector machines	1 (0)	1 (0)	1 (0)	0.9993 (0.0022)	0.9967 (0.0039)	0.9927 (0.0072)	0.8759 (0.0835)	0.9972 (0.0028)
K-nearest neighbors	1 (0)	0.9999 (0.0002)	1 (0)	1 (0)	0.9981 (0.0039)	0.9983 (0.0031)	0.9899 (0.0140)	0.9956 (0.0077)
Naive Bayes	0.9948 (0.0037)	0.9908 (0.0078)	0.9757 (0.0269)	0.9743 (0.0202)	0.9878 (0.0056)	0.9903 (0.0060)	0.9158 (0.0371)	0.9798 (0.0170)
Neural networks	1 (0)	1 (0)	0.9997 (0.0009)	0.9978 (0.0070)	0.9923 (0.0141)	0.9910 (0.0162)	0.9769 (0.0336)	0.9971 (0.0034)

*All approaches are very successful on the given tasks, although K-Nearest Neighbor and Neural Networks appear to be the best performers*.

Table 4 shows that the AUC values for all employed classification approaches are very high (near or equal to the perfect score of 1), suggesting that reliable decisions can be made by using the proposed MPIs. The most difficult task appears to be discerning the stage II patients from stages I and III PD, based on the selected subset of 13 features. However, K-Nearest Neighbor and Neural Network classifiers seem to achieve quite consistent high performance under all tested conditions. Also, using only the 13 features instead of all 84 results in just a slight reduction in performance, providing another evidence in favor of informativeness of the selected MPIs.

### 3.6 Left-Right Side Analysis

Results of the statistical analysis suggest that 14 MPIs out of 84 MPIs in total are relevant for the left-right side analysis between patients and controls (Table 5). Such result indicates that EMG MPIs for grasping, pick and place movements are the most relevant for the left–right side analysis, as well as MPIs extracted from the rotation of the hand movement while the elbow is flexed.

**Table 5:** Relevant MPIs for the left–right side analysis across clinical groups of interest^a^.

Patients/controls	Disease stages (HY)
2 EMG MPIs RH-EE	EMG-VAR RH-EF
2 EMG MPIs RH-EF	ACC MPIs RH-EF
ACC MPIs RH-EF	ACC MPIs GPP-EL
GYRO MPIs RH-EF	GYRO MPIs GPP-EL
EMG MPIs GPP-EL	ACC-RAN MPI GPP-HL
EMG MPIs GPP-HL	GYRO MPIs GPP-HL
	ACC-SSI TT-P

*^a^MPIs are listed in the format MPI movement*.

**Figure 9:**
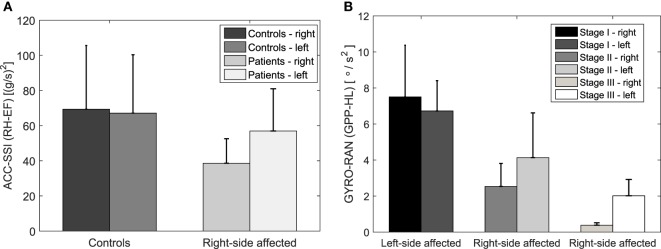
Acc-ssi MPI (RH-EF movement) for controls and right side affected patients **(A)** and Gyro-ran (GPP-HL movement) for different disease stages **(B)**. The mean MPI values for the left and right hand are similar in controls opposite to the patients **(A)**. The mean MPI values decrease from the first to the third stage and their difference between the left- and the right-hand increases **(B)**.

Figure 9A illustrates the mean and standard deviation graph for controls and right-side affected patients for Acc-ssi MPI (RH-EF movement). It can be seen the mean MPI values are almost the same in the case of controls, while in patients, the mean MPI value for the left hand movement is larger than for the right hand movement. Such outcome is expected, since the right side is affected by PD and consequently, has lower performance.

The results of the statistical analysis suggest that 11 MPIs out of 84 MPIs in total are relevant for the left-right side analysis between disease stages (Table 5). It turns out that the ACC and GYRO MPIs for RH-EF, GPP-EL, and GPP-HL are the most common MPIs to evaluate the difference in performance between left and right hand across the disease stages.

Figure 9B illustrates the mean and standard deviation graph across disease stages for Gyro-ran MPI (GPP-HL movement). It can be seen that the mean MPI values decrease from the first to the third stage and their difference between the left- and the right-hand increases. Such result suggests that differences in the performance of the left and right hand become larger with the disease progression. It can be seen that in the case of the left-side affected group (first stage) the MPI values are greater for the right hand. The situation is opposite for the right-side affected group of the second and third disease stage. In both cases, MPI values are greater for the hand less affected by the disease, which is an expected outcome.

### 3.7 Correlations with Clinical Scales

In this section, we want to investigate whether the proposed MPIs are correlated with clinical test and scales. This is particularly important for the possible inclusion of the proposed MPIs into medical protocols. All MPIs that satisfy correlation conditions (explained in the Section 2.4.5) for the tapping test and UPDRS-III scale are listed in Table 6.

**Table 6:** List of MPIs^a^ correlated with tapping test^b^ (*ρ* > 0.5, *p* < 0.05) and UPDRS-III scale^c^ (*ρ* < −0.5, *p* < 0.05).

Tapping test		UPDRS-III scale	
Proximal taping task	Distal taping task	UPDRS-III general	UPDRS-III subscore
EMG MPIs RH-EE L	ACC MPIs RH-EE R	**EMG MPIs RH-EE R L**	**EMG MPIs RH-EE R L**
ACC MPIs RH-EE R	GYRO MPIs GPP-EL R	ACC-RAN RH-EE R	**GYRO MPIs RH-EE R L**
EMG MPIs RH-EF L	**ACC MPIs TT-P R L**	**GYRO MPIs RH-EE R L**	**EMG MPIs RH-EF R L**
ACC MPIs RH-EF R L	**GYRO MPIs TT-P R L**	**EMG MPIs RH-EF R L**	**ACC MPIs RH-EF L**
GYRO MPIs RH-EF L	**ACC MPIs TT-D L**	**ACC MPIs RH-EF L**	**GYRO MPIs RH-EF L**
GYRO MPIs GPP-EL R	**GYRO MPIs TT-D L**	**GYRO MPIs RH-EF L**	**2 EMG MPIs GPP-HL R**
ACC-RAN GPP-HL R		**2 EMG MPIs GPP-HL R**	**GYRO MPIs GPP-HL R**
EMG MPIs TT-P L		ACC-RAN GPP-HL R	**EMG MPIs TT-P L**
**ACC MPIs TT-P R L**		**GYRO MPIs GPP-HL R**	**ACC MPIs TT-P R L**
**GYRO MPIs TT-P R L**		**EMG MPIs TT-P L**	**GYRO MPIs TT-P R L**
**ACC MPIs TT-D R L**		**ACC MPIs TT-P R L**	ACC-RAN TT-D L
**GYRO MPIs TT-D R L**		**GYRO MPIs TT-P L**	GYRO-RAN TT-D L
		GYRO MPIs TT-D L	

*^a^MPIs are listed in the format MPI(s) movement hand (R-right or/and L-left)*.

*^b^MPIs extracted from the tapping test movements (TT-P and TT-D) are correlated with both tapping tasks (bold text)*.

*^c^MPIs correlated with both UPDRS-III scores are marked as bold text*.

**Figure 10:**
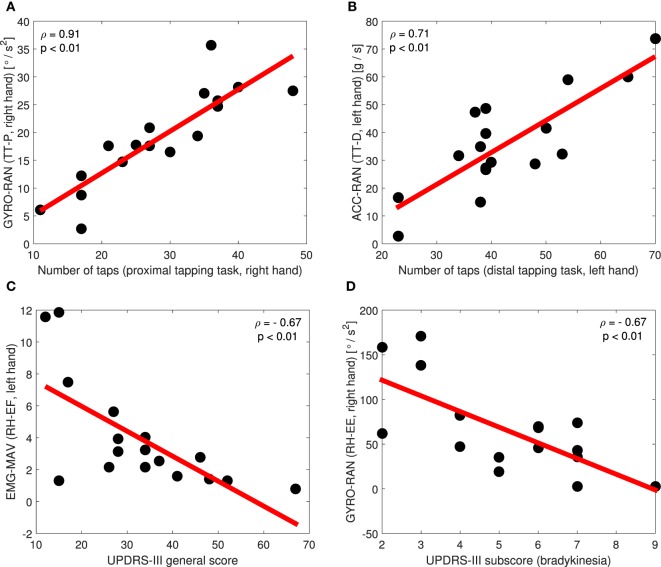
Scatter plots of the correlation between particular MPIs and tapping test **(A,B)**, UPDRS-III general score **(C)**, and UPDRS-III bradykinesia subscore **(D)**.

Scatter plots in Figure 10 illustrate a few examples of the correlation between MPIs and clinical parameters, where the line represents the regression curve. It can be seen that the selected MPIs have a positive correlation with the tapping test (Figures 10A,B), more concretely with the number of taps in two cases of the tapping test (procedure of the tapping test is explained in the Section 2.2). This is expected since the patients who have higher values of MPIs potentially can achieve a larger number of taps within defined time interval (30 s). On the other side, our MPIs have a negative correlation with the UPDRS-III general score (Figure 10C) and subscore for bradykinesya (Figure 10D), since the lower values of our MPIs and higher values on UPDRS-III scale indicate a more severe state of the patient, i.e., more advanced disease stage.

Results of the correlation analysis regarding the tapping test (Table 6) have shown that the most correlated MPIs for both tapping tasks are the ones extracted from the tapping test movements (TT-P and TT-D). Such result is expected, since the same movements are tested during clinical protocol and our sensor measurements. Those MPIs refer to all ACC and GYRO MPIs of both, left- and right-hand movements. In addition to the tapping test movements, ACC and GYRO MPIs from the right-hand RH-EE and GPP-EL movements, as well as from the left hand RH-EF movement have high values of Spearman correlation coefficient *ρ*. MPIs extracted from EMG signals are mostly poorly correlated with tapping test (*ρ* < 0.5, *p* > 0.05), except EMG MPIs in the case of the left-hand RH-EE, RH-EF, and TT-P movements (Table 6).

Results of the correlation analysis regarding the UPDRS-III scale for the general score and bradykinesia subscore highlight mostly the same MPIs in both cases (Table 6). The most correlated MPIs are the ones extracted from the rotation of the hand movements (RH-EE and RH-EF), Table 6. In addition to the rotation of the hand movements, the MPIs from right hand GPP-HL and TT-P movements, as well as MPIs from the left TT-P and TT-D movements have high (absolute) values of Spearman correlation coefficient *ρ*. Since higher values of *ρ* indicate better correlation, those MPIs are very good in terms of correlation with UPDRS-III scale.

### 3.8 Summary

Table 7 summarizes the importance of the MPIs and tested movements across nine criterions of clinical interest. Gyro-ssi and Gyro-ran MPIs are relevant according to all criterions. Particular EMG MPIs are important for the classification aspect and left–right side analysis (both conditions—patients vs. controls and disease stages), while the ACC MPIs are of interest for the left–right side analysis and correlation with clinical scales. Among tested movements, object grasping, pick, and place (both variations—easy and heavy load) turn out to be the most relevant for listed clinical aspects. Reliability analysis has demonstrated the high reliability for all proposed MPIs across all movements (Table 7).

**Table 7:** Importance of the MPIs and tested movements across criterions of clinical interest.

		MPIs							Movement (left and right hand)					
	Criterion	EMG			ACC		GYRO		RH		GPP		TT	
		mav	var	wc	ssi	ran	ssi	ran	EE	EF	EL	HL	P	D
1.	Reliability	✓	✓	✓	✓	✓	✓	✓	✓	✓	✓	✓	✓	✓
2.	Classification patients-controls LDA	✓					✓	✓			✓	✓	✓	✓
3.	Classification patients-controls LASSO	✓					✓	✓		✓	✓	✓	✓	✓
4.	Classification disease stages LDA	✓					✓	✓	✓	✓	✓	✓	✓	✓
5.	Classification disease stages LASSO	✓					✓	✓	✓	✓	✓	✓	✓	✓
6.	Left-right side analysis patients-controls	✓	✓	✓	✓	✓	✓	✓	✓	✓	✓	✓		
7.	Left-right side analysis disease stages		✓		✓	✓	✓	✓		✓	✓	✓	✓	
8.	Correlation—tapping test				✓	✓	✓	✓	✓	✓	✓		✓	✓
9.	Correlation—UPDRS-III	✓	✓	✓	✓	✓	✓	✓	✓	✓		✓	✓	

## 4 Discussion and Conclusion

In recent studies, the use of an armband device has been considered for medical and rehabilitation applications, especially for physiotherapy healthcare (27) and recovery after the stroke (28). The authors in Ref. (27) use MYO Diagnostics application for medical diagnosis and to understand how comfortable subjects feel while performing the movements using the armband device. The study (28) proposes a low-cost rehabilitation system for recovery after the stroke, which consists of an armband device and a data glove. The authors present just the concept of a rehabilitation system based on the virtual environment and gaming to enhance the patient’s motivation. Both studies (27, 28) lack the signal processing, feature extraction analysis, and decision-making procedure behind the interface.

In Ref. (29) the authors propose a multi-sensory gesture-based occupational therapy system, which consists of a Kinect v2, a Leap motion sensor and a Myo armband device. The system is intended to support the everyday activities in the home environment and to encourage the patients to practice and obtain the feedback about their movement performance during usual daily routines. Again, as in Ref. (27, 28) only the concept of the system is presented, along with the general implementation details.

Lack of the sensor signal analysis and processing toward the extraction of the meaningful signal features, as well as the development of the clinically-oriented approaches based on the sensor movement data, are the main drawbacks of the related studies. We have used a wireless armband sensor to acquire arm/hand movements defined by the PD protocol. We propose a set of 84 Movement Performance Indicators (MPIs) to characterize acquired movements. We conducted a thorough analysis of the properties of these MPIs, to identify their importance in terms of relevant clinical aspects (Table 7): (i) reliability; (ii) classification between patients and controls and between disease stages (support to diagnosis and monitoring, respectively); (iii) left–right side analysis between controls and patients, as well as between disease stage groups; and (iv) correlation with clinical scales (tapping test and UPDRS-III). The overall conclusion is that Gyro-ssi and Gyro-ran MPIs are relevant according to all clinically relevant criterions. Particular EMG MPIs are important for the classification aspect and left–right side analysis, while the ACC MPIs are of interest for the left–right side analysis and correlation with clinical scales.

This study complements our previous research (23) with an approach for quantitative movement analysis, based on the arm/hand movement data acquired with an EMG sensor. Our results show that the proposed approach has the potential to be adopted by therapists, to enhance objectivity and precision, during the diagnosis/monitoring evaluations and bradykinesia assessment. At the same time, it opens the possibility of the low-cost assessment tool for patients with the mild to moderate PD stages (I–III according to the modified HY clinical scale).

The armband electromyographic sensor is worn on the forearm and collects the data from the four groups of muscles—flexors, extensors, internal, and external forearm muscles (Section 2.2, Figure 2). One very important conclusion is that external forearm muscles of both hands in PD patients have demonstrated the lowest performance of all forearm muscles in the sense of the muscle activity compared with a control group. This result suggests that external forearm muscles are the most affected by the Parkinson’s disease. Such result is derived from our sensor data but requires additional clinical testing and confirmation.

In the Parkinson’s disease, one side of the body is more affected than the other. Furthermore, the first symptoms of the disease are observed on a particular body side. Along with the disease progress, both sides become affected, but the side on which PD symptoms were first detected, is always affected more. The quantitative assessment of the difference between left and right side of the body would be significant information for the neurologists, since they cannot evaluate it directly or using subjective clinical scales. Consequently, we investigated the differences in the movement performance with left and right hand, relying on the proposed MPIs. Our finding is that those differences are negligible in control subjects, while they can become quite large for Parkinson’s patients, depending on the disease stage.

Collected sensor data in the context of designed MPIs have revealed the bradykinesia patterns in patient movement data. The slowness of the movement and sequential drop of the amplitude over time (so-called “sequence effect”) are visible from the MPIs temporal evolution. Such results indicate the potential of our proposed MPIs to be used by therapists for quantitative assessment of bradykinesia.

Finally, we conclude that sensor data collected from the wireless armband device successfully addressed the same set of relevant aspects in PD like the sensor glove data in our previous research (23). Even more, in this study, we have performed the left–right side analysis, which is not feasible with the sensor glove data, due to its right-hand design. Consequently, our results suggest that the wireless armband sensor can be a possible alternative for high-cost data glove that we used in our previous research. However, the experimental setup, tested movements and extracted Movement Performance Indicators (MPIs) are different in accordance with sensor choice. The advantage of the sensor glove data over the armband device is the quantification of the fine finger movements.

One limitation of the study is the collection of the sensor measurements during the ON-stage only. It would be worth to investigate the movement data characteristics during OFF-stage. The number of subjects and tested movements could be extended in the future. Finally, MPIs proposed in this study are the result of the signal processing in the time domain. Additional MPIs could be extracted from the frequency domain of the sensor signals.

In the future work, we will focus on another important aspect of Parkinson’s disease—balance and stability. We are considering using a low-cost device with sensors of pressure for balance quantification. Furthermore, we plan to test our system on patients recovering from the stroke.

## Ethics Statement

This study was carried out in accordance with the recommendations of the Declaration of Helsinki and the Ethics Committee of the Medical Faculty of Military Medical Academy, University of Defence (Belgrade, Serbia) approved the present study. After the experimental procedures were explained, all subjects signed written informed consent forms.

## Author Contributions

SS, TI, and JS-V designed the study; SS and TI collected the data; SS processed the data; SS and IS analyzed the data; SS wrote the manuscript; and TI, IS, VP, AR, and JS-V revised the manuscript.

## Conflict of Interest Statement

The authors declare that the research was conducted in the absence of any commercial or financial relationships that could be construed as a potential conflict of interest.

## Footnotes

^1^https://www.myo.com/.

^2^Statistics are shown as *mean* ± *SD*.

^3^Parkinson’s disease stadium according to Hoehn & Yahr clinical scale (1).

^4^Evaluation of the motor performance according to the Unified Parkinson’s Disease Rating Scale, section III—motor scores (2).
